# The Frequent Sampling of Wound Scratch Assay Reveals the “Opportunity” Window for Quantitative Evaluation of Cell Motility-Impeding Drugs

**DOI:** 10.3389/fcell.2021.640972

**Published:** 2021-03-11

**Authors:** Sholpan Kauanova, Arshat Urazbayev, Ivan Vorobjev

**Affiliations:** ^1^School of Science and Humanities, Nazarbayev University, Nur-Sultan, Kazakhstan; ^2^National Laboratory Astana, Nazarbayev University, Nur-Sultan, Kazakhstan

**Keywords:** wound healing, high-throughput, label-free microscopy, live-cell imaging, segmentation automation, cell motility

## Abstract

Wound healing assay performed with automated microscopy is widely used in drug testing, cancer cell analysis, and similar approaches. It is easy to perform, and the results are reproducible. However, it is usually used as a semi-quantitative approach because of inefficient image segmentation in transmitted light microscopy. Recently, several algorithms for wound healing quantification were suggested, but none of them was tested on a large dataset. In the current study, we develop a pipeline allowing to achieve correct segmentation of the wound edges in >95% of pictures and extended statistical data processing to eliminate errors of cell culture artifacts. Using this tool, we collected data on wound healing dynamics of 10 cell lines with 10 min time resolution. We determine that the overall kinetics of wound healing is non-linear; however, all cell lines demonstrate linear wound closure dynamics in a 6-h window between the fifth and 12th hours after scratching. We next analyzed microtubule-inhibiting drugs’, nocodazole, vinorelbine, and Taxol, action on the kinetics of wound healing in the drug concentration-dependent way. Within this time window, the measurements of velocity of the cell edge allow the detection of statistically significant data when changes did not exceed 10–15%. All cell lines show decrease in the wound healing velocity at millimolar concentrations of microtubule inhibitors. However, dose-dependent response was cell line specific and drug specific. Cell motility was completely inhibited (edge velocity decreased 100%), while in others, it decreased only slightly (not more than 50%). Nanomolar doses (10–100 nM) of microtubule inhibitors in some cases even elevated cell motility. We speculate that anti-microtubule drugs might have specific effects on cell motility not related to the inhibition of the dynamic instability of microtubules.

## Introduction

Cell migration is essential for many physiological processes including embryonic development, wound repair, angiogenesis, and tumor metastasis (Bindschadler and McGrath, 2007; Friedl and Gilmour, 2009). Cell migration largely depends on the actin cytoskeleton (Ridley et al., 2003), and directional migration of fibroblasts and tumor cells depends on microtubules (Liao et al., 1995).

Primary fibroblast migration is impeded dramatically when microtubules (MT) are completely depolymerized by microtubule-interfering drugs (Vasiliev et al., 1970). The low concentrations of MT inhibitors are sufficient only for the inhibition of dynamic instability of MTs and do not induce their depolymerization; nonetheless, they effectively inhibited certain cell migration (Liao et al., 1995; Vacca et al., 1999; Yang et al., 2010). The overall conclusion from the studies on the effect of MT inhibitors is that the dynamic microtubules are required for cell motility, and in certain cases, it is even not related to the ability of drugs to inhibit cell division (Ngan et al., 2001; Ganguly et al., 2010). MTs are thought to be involved in cell motility through the regulation of Rho GTPases via several pathways (Kaverina and Straube, 2011), facilitating focal adhesion turnover (Kaverina et al., 1999) and providing directional delivery of different cargoes like Golgi-derived vesicles, endosomes, etc., by kinesins to the lamellae (Schmidt et al., 2009; Kaverina and Straube, 2011; Fife et al., 2014).

Microtubule inhibitors from taxanes and vinca alkaloids groups are widely used in clinical practice (Gascoigne and Taylor, 2009; Perez, 2009; Fanale et al., 2015), and the detailed analysis of the behavior of tumor cells under the action of these drugs *in vitro* is of great interest. However, comprehensive analysis of the involvement of MTs in cell motility gives contradictory results. Some cells become nearly immotile under the action of MT inhibitors applied in nanomolar doses (Liao et al., 1995; Ganguly et al., 2015). Other types of cells can move yet at lower speed when MT dynamic is suppressed by low doses of anti-MT drugs and continue moving even in the absence of MTs (Ganguly et al., 2012).

Quantitative data on the concentration-dependent inhibition of cell motility by anti-MT drugs is scarce in the literature. Usually, authors simply make statements about the effect of few concentrations of different drugs.

Obtaining statistically significant results on cell migration requires time-consuming analysis of hundreds of images (Wollman and Stuurman, 2007; Stamm et al., 2016). The most reliable and convenient method of cell motility studies is wound healing assay (Stokes et al., 1991; Codling et al., 2008; Hulkower and Herber, 2011; Kramer et al., 2013; Stamm et al., 2016). However, wound healing analysis is usually made in the semi-quantitative manner, and results are compared after large time intervals (Wang et al., 2019). Software tools increasing quantitative output through automated image segmentation were suggested (Gebäck et al., 2009; Baecker, 2012; Eliceiri et al., 2012; Cortesi et al., 2017), but up to date, none of them becomes widely used for high-throughput study of wound healing due to the necessity to adjust parameters manually. Thus, the researchers still have to develop custom tools for automated image segmentation in a particular experimental setup (Bindschadler and McGrath, 2007; Topman et al., 2012).

In the current study, we developed a pipeline for automated image segmentation with a minimal set of adjustable parameters. It was tested for the accuracy and reproducibility and then used to analyze dose-dependent effects of the three widely used microtubule inhibitors, namely nocodazole, Taxol, and vinorelbine (Jordan and Wilson, 2004; Stanton et al., 2011). While nocodazole and vinorelbine act as microtubule-depolymerizing agents and paclitaxel—as microtubule stabilizer, all three drugs share one common feature—inhibition of the dynamic instability of MTs that is achieved for their nanomolar concentrations (Grigoriev et al., 1999; Jordan and Wilson, 2004).

Using high-throughput analysis, we determined the temporal window when wound healing occurs as a linear process. Linear approximation allowed us to determine relatively small changes under the action of drugs at low concentrations. We found that a dose-dependent response to MT inhibitors becomes significant only for some cultures and always at concentrations exceeding minimal mitostatic ones, and the effect of micromolar concentrations of these drugs is very different between cell lines examined.

## Materials and Methods

### Cell Culture and Live Cell Imaging

Experiments were performed on 3T3, human-derived primary skin fibroblasts (HPFs) donated by Dr. Bolat Sultankuov, HaCaT, A549, HeLa, PC-3, HT1080, and U2OS cell lines. MCF-7 cells with ABC cassette causing epithelial-to-mesenchymal transition after treatment by doxycycline (MCF-7 DOX) were kindly donated by Prof. Eugene Tulchinsky. Cells were maintained according to the regular recommendation of the ATCC for mammalian cultures on a commercially available media DMEM (Sigma, Cat. No. D6546), DMEM/F12 (Sigma, Cat. No. D6421), and L-15 (Sigma, Cat. No. L1518) supplemented with 5% (and 10% for HPF) of Fetal Bovine Serum (Sigma, Cat. No. F2442), and 4–8 nM of L-glutamine (Sigma, Cat. No. G7513). Nocodazole (Sigma, Cat. No. M1404), Taxol (Sigma, Cat. No. T7191), and vinorelbine (Sigma, Cat. No. V2264) were used to model the microtubule turnover inhibition. Mitomycin C (MMC) was used as a model of proliferation deficiency (Sigma, Cat. No. M4287). Doxycycline (DOX) was used to induce the epithelial-to-mesenchymal transition in the MCF-7 cell line (Sigma, Cat. No. 324385).

For a wound assay, cells yielded from 25 cm^2^ flask grown to 100% cell confluency (∼2.8–3.0 × 10^6^) were seeded into 24-well culture plate (Sigma, Cat. No. Z707791) or eight-well NUNC II cover glass chambered system (Sigma, Cat. No. TMO155411). Cells were seeded at 5.8 × 10^4^ per well of a 24-well plate and 3.5 × 10^6^ for an eight-well glass chamber and cultivated for 36 h to achieve complete spreading of cells and 100% of monolayer confluency for cancer and normal cells and up to 72 h for primary human skin fibroblasts. The monolayer wounding was performed after ∼60 min of incubation with drugs in CO_2_-independent media (in heating or at microscope climate chamber). Gentle cross scratching with a 200-μl tip or polished toothpick sterilized with ethanol was applied to every well. For microscopic imaging, CO_2_-independent, HEPES free, DMEM (Thermo Fisher, Cat. No.18045054) supplemented by regular FBS and complemented with drugs was used for long-term exposition on a heating stage of a microscope.

Glass-bottomed Nunc LabTech II slides were used to substitute the plastic surface in similar imaging conditions. A study of MMC treatment was performed on eight-well glass chambers with nocodazole 300 nM used as the positive control.

Experiments with nocodazole, Taxol, and vinorelbine were performed with serial dilutions of the drugs in concentrations descending from 3,000 to 10 nM in 24-well plates. The procedure is provided in Supplementary File S1. For several cultures, additional experiments with lower doses of drugs (down to 0.3 nM) were performed. Nocodazole, Taxol, and vinorelbine titration was performed simultaneously with control wells within the 24-well plate. The imaging was started immediately after wound application and continued for 24 h. The microscope programming for ∼20–30 min was performed to set the coordinates of four fields of view within each well in the 24-well plate. Controls are triplicated in each plate, and three to four independent experiments were performed for each cell line and treatment.

Imaging was performed under 10/0.25× magnification of a Planachromate objective on an Axio Observer Z1 microscope equipped with a heating chamber, ORCA V2 16-bit sCMOS camera, and under control of ZEN software allowing the automated time-lapse collection from the multiple stage positions in one experiment. The microscope pre-heating was performed for 4 h, illumination adjustment was performed before the plate was inserted to the microscope stage, and acquisition positions were set under control of the camera immediately after scratch was applied. These data were further processed for building a raw dataset of wound closure dynamics.

### The Wound Contour Computer Segmentation

To segment the time-lapse records of closing wounds, we developed the MatLab tool. It reads the file content of the directory with images organized into a library containing a separate folder of individual time-lapse records (as TIFF, JPEG, or PNG sequence). The total number of images in the library was ∼200,000. Each image is uploaded to the RAM sequentially to render an output, and then, it immediately recorded on the storage location in the form of a binary image with a mask of the wound gap area. The detailed information of the procedure can be found in Supplementary File S2. The tool and used user manual are uploaded in https://github.com/Sholpan-Kauanova/HTM_Wound_Healing_Tool.

**FIGURE 1:**
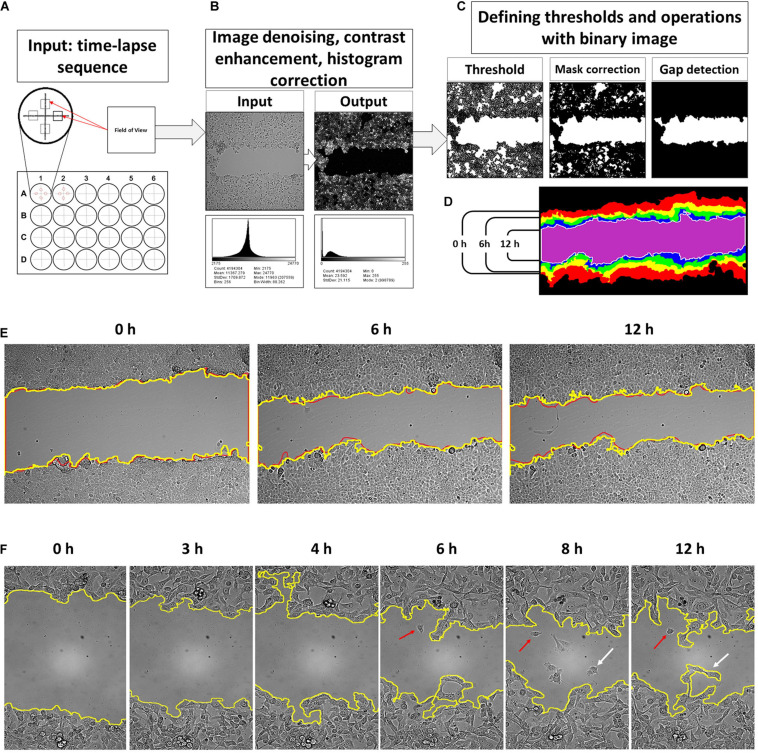
An input for a developed tool is time-lapse sequences collected from four fields of view of each well of a multi-well plate **(A)**. The software processes consequently each input grayscale image and applies image correction procedure where noise filtering and histogram reassignment are performed **(B)**. Then, the script applies a threshold on the filtered grayscale image to convert it into a binary form for correction and gap detection procedure **(C)**. The obtained binary masks are then collected into a sequence of wound contours and area counts **(D)**. The results of the contour segmentation accuracy were compared with manually drawn overlays. The difference between manually outlined and segmented by algorithm gaps was, on average, ∼2.09% and was set as error measure **(E)**. Detailed examination of the obtained gap contour overlays reveals that there are groups of cells moving faster than the neighbor lattice for several cell lines. This event occurs after 6–12 h of scratch (**F**, red arrows). Such cells start to detach from the cell lattice and may reenter back. It was observed more frequently for HT1080 (enlargement **E**) and 3T3 cells. This process is relatively rare in the case of A549, HaCaT, and MCF-7 cells. However, scripted segmentation includes the area of such cells to the calculated result (**F**, white arrows).

The parameters for the segmentation tool were determined in advance in respect to the average size of the mammalian cell (Stamm et al., 2016). The accuracy was evaluated with the aim of a test set of 876 test images of the 8- and 16-bit depth collected with the EVOS FL (with 5× and 10× magnification objectives) and the Axio Observer (with Zeiss MRM camera with a 10× objective, and ORCAFlashV2 Hamamatsu camera with 10× and 20× objectives). The test sets of images were picked from experiments performed on 24-well plastic plates and 8-well glass-bottom slides (Figure 1A represents a 24-well plate).

The image-processing steps were chosen based on a survey of published methods. The processing consists of noise filtration to enhance the cell contour contrast, then thresholding, and binary mask rendering (Valster et al., 2005; Bindschadler and McGrath, 2007; Gebäck et al., 2009; Bise et al., 2011; Kanade et al., 2011; Zordan et al., 2011; Baecker, 2012; Milde et al., 2012; Johnston et al., 2014; Cardona et al., 2015; Vargas et al., 2016; Cortesi et al., 2017; Molinie and Gautreau, 2018; Main et al., 2020; Suarez-Arnedo et al., 2020). There is flood fill check and recalculation of new thresholds and the binary mask correction procedure to ensure that segmentation of the wound is performed correctly (Valster et al., 2005; Bindschadler and McGrath, 2007; Petrie et al., 2009; Zordan et al., 2011; Milde et al., 2012; Johnston et al., 2014; Vargas et al., 2016; Raudenska et al., 2019).

The core of a tool is filtering sequence to remove the illumination noise and unevenness and enhance contrast stepwise by: (a) image powering, (b) the flat field correction by Gaussians smoothing (σ_1_ = 2, σ_2_ = 1), (c) then applying a custom-built denoising entropy filter that reduces the remnants of illumination artifacts and enhances the contrast of the intermediate image, (d) background subtraction by the “TopHat” filter that reassigns image histogram to a new composition, and (e) the average filter to smooth and subtract small particles (dust, debris, etc.). This procedure results in a grayscale image with histogram composition like in fluorescent microscopy (Bise et al., 2011; Kanade et al., 2011). On this new picture, cells are highlighted, and the background becomes significantly darker (Figure 1B).

The next step is to create a binary mask of the wound gap for segmentation procedure. Thresholding was set to target the pixel values of a gap that are below the histogram mean. So, the thresholding value calculated by dividing the image histogram mean by ***T*** coefficient, which shall be a positive integer higher than 1. The histogram mean depends on the cell line morphology, and the optimal value of the coefficient T was calculated empirically on test image sets of 8- and 16-bit images. For an 8-bit camera, the coefficient was determined to be 2.2, and for the 16-bit camera, it is 3.14. The cell lines producing low-contrast images such as 3T3 and HPF requires the coefficient to be 2.2 for a 16-bit camera and 1.2 for an 8-bit camera. In all cases, the coefficient can be set as >31% of the histogram for a high-contrast cancer cell and >45% of a histogram mean of low contrast 3T3 and HPF cells to produce an accurate binary mask with the least amount of misrecognized parts of monolayer and gap. We further exploit the notable feature of the scratch assay, which is the presence of the empty area in a middle of the field of view. There is a loop for a conditional operator that is introduced to check whether an image contains an empty cleft in the middle. Then the binary image contours were smoothed using the following procedures: (1) binary closure, (2) dilatation, (3) closure, and (4) erosion with kernel size of minimal radii to avoid excessive mask degradation. It produces a solid mask of the gap without isthmuses of the contour.

Thus, gap detection is performed by selecting the biggest blob of pixels in a binary image located at the center of the image (Figure 1C). The perimeter of the detected gap is then outlined, and thus, wound contours are obtained (Figure 1D). If the gap was closed completely, and there is nothing to detect, the script anyway will create an empty image.

### The Segmentation Accuracy and Efficiency Validation

Validation of the gap segmentation accuracy was performed by comparison of the area of the binary mask versus manually outlined gaps (Figure 1E). The difference of the masks outlined manually and those generated by a computer using the script developed was set as an error percentage. The average difference between the wound areas at manually outlined and computer-segmented images was ∼2.56% (SD = 2.09, *n* = 219) (Figure 1E).

Image segmentation was further compared with the results of the FIJI/ImageJ MRI Wound healing tool on the set of randomly chosen images (total 987 images, containing randomly chosen 8- and 16-bit images, collected with the objectives 10× and 20×, for wound healing performed on plastic and glass substrates). The result was evaluated by comparison of the outline masks obtained after segmentation by FIJI and MatLab tool. The masks were overlayed on the original images. The filtering settings for the FIJI macros were restricted to be the same for all time-lapse series, similarly as it was performed in our MatLab script. FIJI had only about >50% of the gaps in the set images that were segmented correctly (i.e., gap and its margin was visually coinciding with the outline mask). Our MatLab tool shows ∼95% detection accuracy for the gap and its borders on all 987 images. MatLab-based software failed in the detecting borders correctly for only ∼5% of images. These images usually included artifacts such as floating debris and crawling cells (Figure 1F, arrows).

The segmentation efficiency evaluation was performed further on a bigger experimental set to confirm the error. It was performed by an experienced user comparing the computer-segmented gap mask with the wound contour on the original image. Segmentation was considered as failed when there was significant visual mismatch between the segmentation contour and visual contour of the wound boundary. On average, the number of such images did not exceed 5% for 24 h. These segmentation errors distributed unequally for the image sets studied. The error frequency increases with time after scratching, and misrecognized boundaries were observed mainly 15 and more hours after scratching. It coincides with the changes in cell morphology when cells on the edge spread completely, and the wound margin became dim.

### The Bias Factors in Detection of the Wound Gap

After scratching of the monolayer, the cells start to advance directly into the empty gap area. The migrating cells have different rate of spreading and velocity of movement, and after several hours, the monolayer lattice becomes irregular with the perimeter bending significantly. Some cells depart from the leading edge into the wound area (Figure 1F, arrows). These cells either continue migrating away from the lattice or return and join it. The frequency of such events increases after 6 h. Such events are frequent for HT1080 (Figure 1F), less common for 3T3, PC-3, U2OS, and MCF-7 cell lines, and relatively rare for A549 (Figure 1E), HaCaT, HeLa, and HPF.

Estimation of the impact of such cells to wound closure is challenging, and gap measurements become inaccurate. It introduces an analysis constraint of 12 h time interval when such events remain relatively rare for all cell lines used in our study. The second constraint is the dramatic difference of the velocity along the wound edge (Figure 1D), which makes gap width highly non-uniform. Gap measurements become rich for outliers decreasing the analysis sensitivity (Wang et al., 2019). To overcome the problem, sampling frequency could be increased for a number of measurements of each time point and by decreasing the time intervals too. We collected the whole gap area change within the field of view as the measure of cell edge expansion. The mean displacement was calculated, and low-pass data filtering was performed by averaging data in time by *N* = 6 with a sampling interval of 10 min for a 1-h smoothing. The absolute area displacement (DT) of the cell lattice expresses expansion of the monolayer on a 2D surface. With respect to the n^*th*^ frame of time-lapse, the monolayer displacement to the initial time point was obtained as DT = |T_0_ − T_*n*_|. Then, DT is converted to metric values according to the pixel size of the image generated on a camera chip.

### Wound Healing Velocity Calculations

To calculate the mean velocity of wound edges, the displacement of the area between sequential data points is divided by the length of the wound. Then it is divided by 2, to determine the average speed of cells at each side of wound.

### Determining the Minimal Mitostatic Concentration

The minimal mitostatic concentration was defined by counting the dividing cells in a monolayer at the edges of the experimental wound. The rounded up cells were followed on the time-lapse sequences. The MT drugs increase the average duration of mitosis and thus increasing the quantity of the rounded up cell compared with the control. The treatment doses where percentage of the rounded up cells (counted in 10 fields of view) was > 3 times bigger than in the control were considered as causing severe mitostatic effect.

### Image Processing and Data Analysis Environment

Images were obtained and exported to TIFF with the ZEN 2.3 software. The image-processing script is compatible with MatLab version 2014 and higher, and requires Simulink, Image Processing Toolbox, and Mapping toolbox. The FIJI/ImageJ was used to manually process and compare the images after the segmentation was performed in MatLab, and to montage the figures. GraphPad ver.5 was used to perform the statistical analysis and montage the figures. To compare the effect, we applied one-way ANOVA, two-way ANOVA, and *t*-test. Krita was used to edit and annotate the figures. See Supplementary File S3 for complete information on software tool used.

## Results

### Wound Closure Dynamics

**FIGURE 2:**
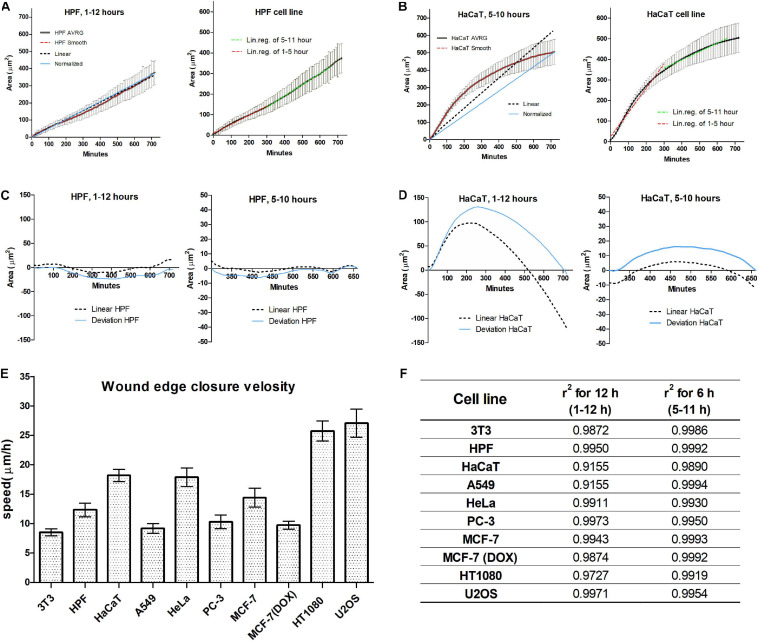
Wound closure of the human-derived primary skin fibroblast (HPF) cell line occurs during 12 h with a constant speed **(A)**; the healing curve shows a good fit to linear and normalized curves **(C)**. HaCaT cell line shows non-linear closure **(B)**; however, piecewise analysis shows that there is a 6-h interval with good linearity **(D)**. Within this 6-h long interval, all cell lines show a good linear fit, compared to a 12-h long period **(F)**. Within this time interval, we determined the speed is to be dependent on the cell line, and values are similar for normal and cancer cells, except for the HT1080 and U2OS cell lines **(E)**.

Wound closure dynamics depends on the cell line; two closure patterns were observed. In the first pattern, the wound closure appears with a nearly constant rate from the first hour after scratching. This pattern was observed for 3T3, MCF-7, PC-3, and for human primary fibroblasts (Figure 2A). In the second pattern, wound closure speed decreases with time for several hours after scratching, and the overall process is significantly non-linear for A549, HT1080, and HaCaT (Figure 2B). In this case, the linear regression fit passing through the origin deviates significantly from the best fit line creating uncertainty in speed measurement. The piecewise analysis of non-linear curves shows that the rate of wound closure in this case is decelerating with time, and linear regression could be applied to the part of the overall curve (5–11 h) with significant precision (Figures 2C,D). In this interval, non-linearity of wound healing process for all cultures examined becomes small (*r*^2^ > 0.985), and it is possible to identify the treatment effect with high precision.

We further compared wound healing process characterized by the average velocity of the advancement of the cell edge during the time interval 5–11 h after scratching. The highest velocity was observed for U2OS (27.08 ± 4.50 μm/h) and HT1080 (25.75 ± 3.21 μm/h) cell lines, while 3T3 (12.32 ± 2.19 μm/h) and A549 (9.17 ± 0.97 μm/h) had the smallest speed of edge advancing. The overall range of wound healing is in accord with previously published data (Wang et al., 2019).

It is interesting to notice that MCF-7 cells with nearly cobblestone morphology have nearly two times faster speed (14.41 ± 2.53 μm/h) compared with MCF-7 cells after doxycycline-stimulated EMT (9.71 ± 1.26 μm/h).

### The Adhesiveness of Substrate Surface Promotes the Wound Closure Dynamics

The adhesive properties of the surface have an effect on cell motility (DiMilla et al., 1991; Huang and Ingber, 1999; Kim and Wirtz, 2013). To be able to generalize our results of the action of microtubule inhibitors, the comparison of wound healing process on tissue culture plastic and less adhesive borosilicate glass was performed. We utilize the limited attachment ability of cells to the uncoated glass to study the migratory potential inhibition/promotion. Our preliminary experiments using poly-L-lysin coating show no difference compared with plastic on cell motility for several cancer cell lines (data not shown).

The pattern of closure (linear or non-linear) of the wounds on the glass surface remains similar to that observed on the plastic surface, while cell spreading on the glass surface was significantly slower (data not shown). During the first 12 h after scratching A549, HT1080, HaCaT, and MCF-7 cells demonstrated non-linear wound closure kinetics, while gap closure was linear for 3T3, HPF, and doxycycline-treated MCF-7 cells. All cell lines studied show good linearity at 5- to 11-h intervals.

All cells except HeLa moved on the borosilicate glass similarly or faster than on the highly adhesive plastic surface. The velocity of HeLa cells significantly decreased on glass (∼30%), and the cells remained less spread compared with those on the plastic surface. The wound closure rate measured on the interval of 5–11 h shows no significant difference of wound closure speed on glass for human skin fibroblasts (Table 1). The significantly decreased rate of wound closure (>25%) on plastic was observed for 3T3 and A549 cell lines. For MCF-7, the significant decrease on plastic was observed only for doxycycline-treated cells, wherein the difference was ∼18% (Table 1).

**TABLE 1:** The wound edge velocity on plastic and glass surface.

Cell line	Velocity (μm/h)		Ratio (%)	*p*-value
	Plastic	Glass		
3T3	8.49	11.58	73.40*	*p* < 0.001
HPF	12.33	12.9	95.56	ns, *p* = 0.0876
HaCaT	18.18	19.55	93.02	ns, *p* = 0.3638
A549	9.17	16.66	55.07*	*p* < 0.001
HeLa	17.88	12.29	145.51*	*p* < 0.001
PC-3	10.29	N/A^∗∗^	N/A^∗∗^	N/A^∗∗^
MCF-7	14.41	16.61	86.76	ns, *p* = 0.1983
MCF DOX	9.72	11.76	82.63*	*p* < 0.0001
HT1080	25.76	26.54	97.04	ns, *p* = 0.7457
U2OS	27.09	27.78	97.49	ns, *p* = 0.1648

All differences marked by (*) are highly significant, *p* < 0.001. **PC-3 cells are not capable of spreading and forming a solid lattice, which is required for wound healing assay.

The notable exception was the behavior of the PC-3 cell line. After seeding on the glass surface, the PC-3 cells remained non-spread and stayed nearly immotile for 24 h, with a small fraction of highly mobile individually migrating cells even in the control. Thus, it is impossible to follow wound closure dynamics for PC-3 cells on the glass surface.

The comparison of cell motility on the plastic and glass surfaces shows discordant results. The motility of 3T3, A549, and doxycycline-treated MCF-7 cells on the glass surface was significantly faster, for HPF, HaCaT, MCF-7, HT1080, and U2OS. The difference was insignificant, while HeLa cells moved significantly slower (Table 1). Thus, further drug titration experiments were performed in 24-well plates with plastic surface.

### Effect of Microtubule Inhibitors on Wound Closure

Depolymerizing drugs nocodazole and vinorelbine applied in micromolar concentrations led to depolymerization of microtubules, while at nanomolar (100–300 nM) concentrations, they usually have insignificant effect on the spatial distribution of microtubules but suppress dynamic instability of the MT plus ends (Grigoriev et al., 1999; Matov et al., 2010). The effects of Taxol are somehow different—complete stabilization of MTs along with disorganization of the radial array is achieved at micromolar concentrations (1–3 μM), while moderate rearrangement of the MT array along with inhibition of the dynamic instability is achieved at nanomolar doses (100–300 nM) (Wang et al., 2003).

More detailed analysis shows that the minimal effective concentrations of all three drugs for inhibiting MT plus-end growth are 10–30 nM for 3T3, HT1080, and A549 cells (Serikbaeva et al., 2018). Thus, we titrated all inhibitors in wound healing experiments in the range of concentrations from 1 nM to 1–3 μM.

Two questions were addressed during the analysis of the action of MT inhibitors: (i) Does complete depolymerization of MTs in the interphase cells caused by micromolar concentrations (1–3 μM) of nocodazole and vinorelbine completely inhibit cell motility? (ii) Do nanomolar doses (10–300 nM) of MT inhibitor demonstrate effects on cell motility too?

**FIGURE 3:**
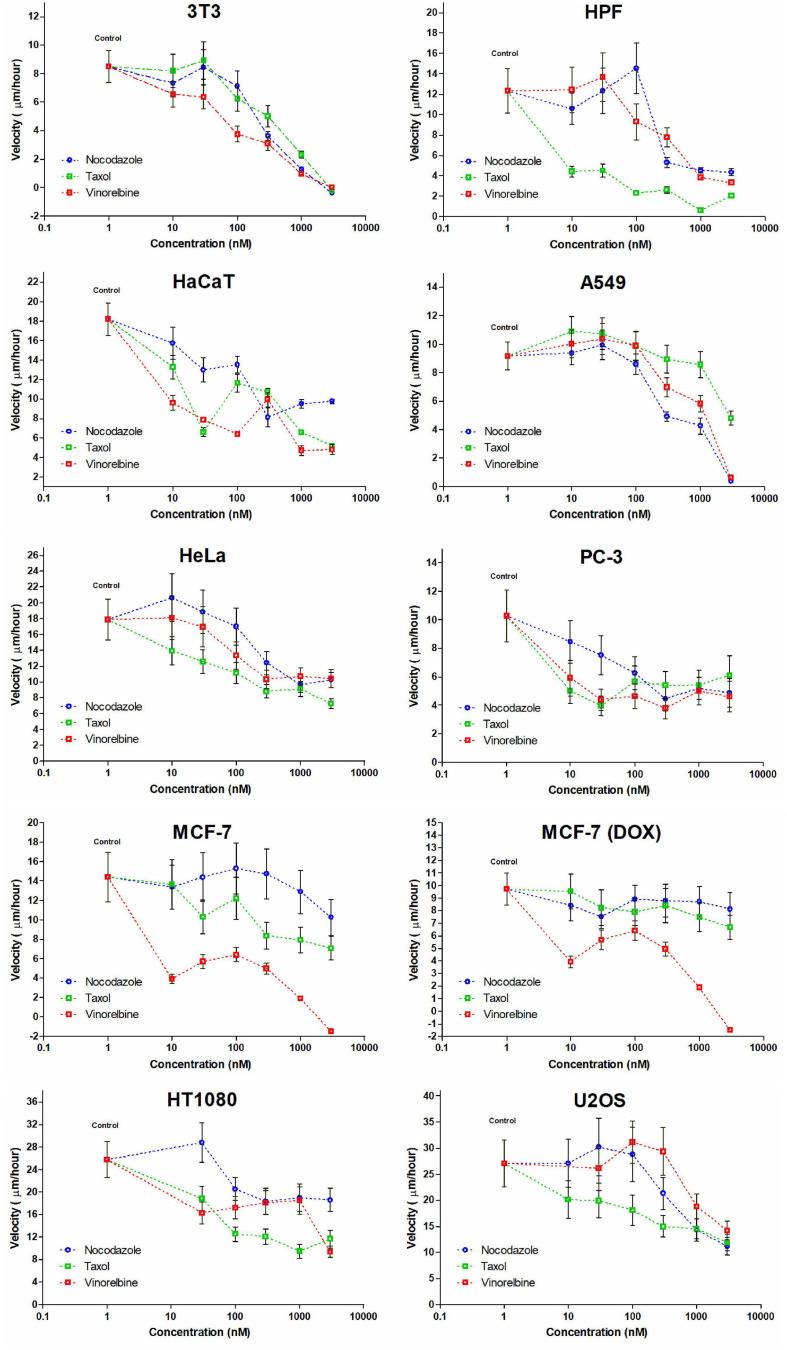
Dose-dependent effect appears not evenly for cell lines studied and IC50 estimations impossible for some cultures. Only in few cases, a decrease in cell velocity is monotonic (for HaCaT and A549 cells under treatment by nocodazole, HeLa, and U2OS cells with similar gradual response for Taxol and for 3T3 cells under treatment by vinorelbine).

All MT drugs applied at concentrations of 1–3 μM slowdown cell motility in all cell cultures. However, the complete stop of wound edge was observed only for several cell lines when speeds turned negative after drug treatment (Table 2). The edge velocity decreases unevenly for tested cell cultures, and the dose-dependent effect is non-linear (Figure 3).

**TABLE 2:** The wound edge velocity changes under drug treatment by microtubule-destabilizing drugs.

Cell line	Nocodazole treatment					
	10 nM	30 nM	100 nM	300 nM	1,000 nM	3,000 nM
3T3	86.28**	99.41 ^*ns*^	83.91**	42.61**	15.12**	−4.56**
HPF	85.91 ^*ns*^	100.03 ^*ns*^	117.93*	43.11**	36.90**	35.20**
HaCaT	86.52**	71.42**	74.37**	44.75**	52.30**	53.75**
A549	102.20 ^*ns*^	108.43*	93.81 ^*ns*^	53.90**	46.72**	4.50**
HeLa	115.37*	105.44 ^*ns*^	95.15 ^*ns*^	69.35**	53.97**	57.37**
PC-3	82.36**	73.19*	60.96**	43.44**	50.39**	47.48**
MCF-7	92.71 ^*ns*^	99.90 ^*ns*^	106.05 ^*ns*^	102.10 ^*ns*^	89.43 ^*ns*^	71.17**
MCF DOX	86.71**	77.31**	91.74^*ns*^	90.49**	89.54 ^*ns*^	83.74**
HT1080	N/A	111.76*	79.67*	71.22*	73.73*	72.24*
U2OS	100.15 ^*ns*^	111.48 ^*ns*^	106.24 ^*ns*^	78.82**	52.80**	41.09**

**Cell line**	**Taxol treatment**					
	**10 nM**	**30 nM**	**100 nM**	**300 nM**	**1,000 nM**	**3,000 nM**

3T3	96.37^*ns*^	104.82 ^*ns*^	73.65**	59.07**	27.44**	−2.37**
HPF	35.69**	36.81**	18.57**	21.13**	5.09**	16.65**
HaCaT	73.07**	36.35**	63.99**	58.89**	36.19**	28.62**
A549	118.79**	117.05**	107.39*	97.53 ^*ns*^	93.40*	52.59**
HeLa	77.97**	70.31**	62.39**	49.25**	51.06**	40.22**
PC-3	48.77**	38.58**	55.26**	52.61**	52.95**	59.62**
MCF-7	94.65^*ns*^	71.62**	84.73**	58.07**	55.05**	49.13**
MCF DOX	98.00^*ns*^	84.80**	81.31**	86.56**	77.16**	68.89**
HT1080	N/A	73.27**	48.45**	46.78**	36.73**	45.21**
U2OS	74.37**	73.66**	66.87**	55.31**	53.77**	43.68**

**Cell line**	**Vinorelbine treatment**					
	**10 nM**	**30 nM**	**100 nM**	**300 nM**	**1,000 nM**	**3,000 nM**

3T3	77.22**	74.84**	44.27**	36.45**	11.29**	−0.09**
HPF	100.72 ^*ns*^	110.96 ^*ns*^	75.56**	63.19**	30.95**	26.92**
HaCaT	52.88**	43.36**	35.23**	54.81**	25.75**	26.30**
A549	109.16 *	112.96 *	107.84 *	76.25**	63.70**	6.93**
HeLa	101.28 ^*ns*^	94.76 ^*ns*^	74.71**	57.92**	60.00**	58.26**
PC-3	57.62**	42.69**	45.17**	36.88**	48.68**	44.88**
MCF-7	27.16**	39.47**	44.55**	34.40**	13.08**	−10.35**
MCF DOX	40.27**	58.52**	66.06**	51.01**	19.40**	−15.35**
HT1080	N/A	63.43**	66.93**	70.28**	71.96**	36.23**
U2OS	N/A	96.52**	114.96**	108.37*	69.57**	52.48**

Ns, non-significant. *Significant, *p* < 0.05. **Highly significant, *p* < 0.001 by Mann–Whitney U test.

The non-transformed cells (3T3, HPF, and HaCaT) are somehow sensitive compared with cancer lines. We defined three specific common patterns on cell motility caused by the drugs for mammalian cell cultures. The 3T3 cells completely stopped after these treatments (Figure 3), and the cell edges slightly shrunk after the fifth hour. The motility of human primary fibroblasts derived from skin and HaCaT cells also was significantly inhibited by the micromolar doses of inhibitors.

Cancer cell cultures show notable decrease in velocity (>7 times compared with the control for A549); however, complete inhibition of motility occurred rarely, and the most effective drug was vinorelbine for MCF-7, HPF, and U2OS cells (Table 2). Vinorelbine treatment results in significant slowdown for A549, HaCaT, HT1080, 3T3, PC-3, and HeLa. Nocodazole treatment results in significant slowdown for A549, HT1080, 3T3, PC-3, U2OS, and HeLa. Taxol treatment results in significant slowdown for HaCaT, HT1080, 3T3, PC-3, U2OS, and HeLa, but still, inhibition of motility of these cells was incomplete.

Moderate effect (when slowdown was significant yet <3 times compared with the control) was observed for HT1080 and PC-3 cells for all treatments, for HeLa cells for nocodazole and vinorelbine treatments, for MCF-7 and U2OS cells for vinorelbine and Taxol treatments, for HaCaT only for vinorelbine treatment, and for A549 only for Taxol treatment.

The overall dose–response dependence curve in majority of the cases was non-linear and non-monotonic (Figure 3) precluding determination of IC50 for several cultures. So far, IC50 could be determined only for HaCaT and A549 cells (nocodazole treatment), for HeLa and U2OS cells (Taxol treatment), and for 3T3 cells (vinorelbine treatment) (Figure 3).

Minor effects on cell motility (velocity decrease <50%) were observed for doxycycline-treated MCF-7 cells under nocodazole and Taxol treatments, while sensitivity to vinorelbine of this culture remains similar to untreated MCF-7.

Finally, we observed speed increase under the treatments with nanomolar doses of MT inhibitors (10–100 nM) for 3T3, HPF, A549, HeLa, MCF-7, HT1080, and U2OS. The velocity increase in these cases did not exceed 20%. Nevertheless, significant acceleration of wound healing occurred under nocodazole treatment (HPF, A549, HeLa, and HT1080 cells), vinorelbine treatment (A549 and U2OS cells), and Taxol treatment (A549 cells) (Table 2).

Anti-microtubule drugs are widely used in the cancer treatment in concentrations that inhibit cell division (Fanale et al., 2015). To determine whether minimal mitostatic doses have an effect on cell motility, we examined life histories of cells undergoing mitosis in our experiments. For each cell culture, at least 30 life histories were analyzed for each dose. For all cell lines tested, minimal mitostatic concentration of nocodazole is in the range of 100–300 nM; Taxol begins to show mitostatic effect in the range of 30–100 nM, and vinorelbine impairs normal division in broad range from 10 to 300 nM (Table 3), while severe slowdown of wound edge occurs at higher doses for a given cell line.

**TABLE 3:** Minimal mitostatic concentrations of drugs (in nM), causing significant mitotic delay or blockade.

Cell line	Nocodazole	Taxol	Vinorelbine
3T3	300 nM	100 nM	10 nM
A549	100 nM	300 nM	300 nM
HaCaT	100 nM	30 nM	10 nM
HPF	100 nM	10 nM	30 nM
HT1080	300 nM	100 nM	100 nM
MCF-7	100 nM	30 nM	≤ 10 nM
PC-3	100 nM	30 nM	≤ 10 nM
U2OS	100 nM	30 nM	300 nM
HeLa	100 nM	10 nM	100 nM

*For each cell culture, at least 50 mitoses were analyzed at minimal mitostatic concentration.*

Treatment by Mitomycin C (MMC) was performed as an alternative model of cell division inhibition to evaluate the impact of doubling cells on wound healing. The area displacement analysis shows that MMC treatment caused no dramatic effect on wound closure of most cell lines studied: wound closure decreased insignificantly for all cells examined except HPF (decrease significant—p = 0.03) and HeLa where it insignificantly increased (Table 4).

**TABLE 4:** The wound edge velocity (in μm/h) changes under Mitomycin C treatment.

Cell line	Velocity (μm/h)		Ratio (%)	*p-*value
	Control	MMC		
HPF	31.97	23.14*	72.36	*p* < 0.05
HeLa	36.87	48.35	131.15	ns, *p* = 0.136
PC-3	26.92	23.64	87.82	ns, *p* = 0.515
MCF-7	36.46	35.30	96.79	ns, *p* = 0.885
HT1080	47.50	36.07	75.94	ns, *p* = 0.0765
U2OS	64.58	57.02	88.30	ns, *p* = 0.357

ns, non-significant. *Significant, *p* < 0.05.

**FIGURE 4:**
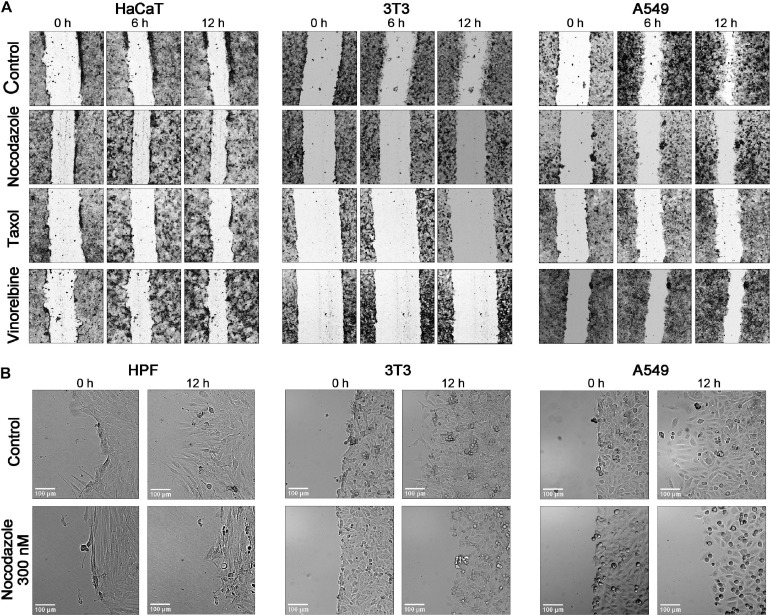
Comparison of the time course of wound closure with the anti-MT drugs in a 12-h interval shows that the untreated cells HaCaT, 3T3, and A549 advance significantly into a wound, while the scratch area is closing slowly in the presence of nocodazole, Taxol, and vinorelbine **(A)**. **(B)** Enlargement of a scene for the wound edge of the control cells and cells treated by nocodazole (300 nM). HPF and 3T3 cells in normal conditions form long lamellae protrusions, while lamellas are significantly shorter after treatment with nocodazole. The A549 shows a similar behavior. After nocodazole treatment, no lamellae appear at the wound edge. Cells arrested in mitosis are evident under nocodazole treatment in all three cultures. Bar = 100 μm (for enlargement).

The cell polarization at the edge of the wound changed under drug treatment, but changes were different among cell lines (Figure 4). Human skin fibroblasts and 3T3 show significant shortening of lamellae under nocodazole 300 nM treatment coinciding with speed decrease, while HaCaT kept their shape like that of the control on doses causing significant slowdown of motility. Cancer cell lines show similar variety of responses, and HT1080 and U2OS remain well spread after treatment with 1–3 μM of a drug.

## Discussion

In this work, for determining dose-dependent effects of microtubule inhibitors on cell motility, we performed a search for an appropriate pipeline. Our method relies on optimization of automated algorithm for the wound gap segmentation and further data processing to extract the statistically significant measurements.

We determined that optimal algorithm must contain the following steps: (i) background correction, (ii) noise filtration, (iii) detection of wound gap, and (iv) contour smoothing by dilation and erosion with small radius. The method provides accuracy similar to that of other algorithms, namely TScratch (Gebäck et al., 2009), AIM (Cortesi et al., 2017), and ImageJ tools (Baecker, 2012; Nunes and Dias, 2017; Suarez-Arnedo et al., 2020). The accuracy of the algorithm was tested on the larger dataset compared with previous methods and allowed us to sustain a recognition accuracy of >95% on a thousand of images recorded in automated mode. The only parameter to be adjusted is threshold, which regulated by coefficient *T*. The search for optimal coefficient was made for all cell lines represented in the study and on test images available for other tools. We defined that the best result is obtained when *T* = 3.14 (or Pi) for images obtained with a 16-bit camera and ∼2.2 for 8-bit cameras. For 3T3 and HPF, the coefficients set 2.2 for 16-bit images and 1.2 for 8-bit. Thus, we recommend starting with *T* = 3.14 when testing cancer cells and try to apply lower values for the cells with thin lamella. The detailed procedure for determining the proper coefficient is presented in Supplementary File S2 and at https://github.com/Sholpan-Kauanova/HTM_Wound_Healing_Tool.

Wound closure is usually described as a linear approximation (Jonkman et al., 2014; Bobadilla et al., 2019). However, the measurements taken at large time intervals do not allow to confirm that advancement of the cell edge occurs with constant speed. Recently, different quantification methods were used for the analysis of cell migration in wound healing assays (Bobadilla et al., 2019). In the most common ones, wound width or area change is measured (Masuzzo et al., 2016; Grada et al., 2017), and the slope of a linear approximation to the change in wound area is determined (Jonkman et al., 2014).

When frequent measurements of wound healing were applied, the process appears to be non-linear. The overall dynamics is approximated by a sigmoid curve (Topman et al., 2012; Masuzzo et al., 2016). The non-linear process is difficult for the detailed experimental analysis since it includes several variables (Potashnikova et al., 2019). Considering that the long slope of the sigmoid curve that describes wound healing is relatively linear (Topman et al., 2012), we addressed this question: Could wound healing be sufficiently approximated by linear regression for a certain period of time?

We selected the velocity obtained from linear approximation of a piece of wound closure curve between the 5th and 12th hours after scratching as a key parameter to analyze the velocity changes with respect to different treatments. To test the internal data consistency, we used to compare SEM and confidence interval (CI). The measured SEM value was at least two times smaller than the predicted 95% CI. Thus, our data show high reliability, and we were allowed to apply the goodness of linear fit (*r*^2^) to determine the time interval where process is highly monotonous (*r*^2^ > 0.99). The analysis reveals that the measured velocity of a wound does not depend on the wound initial width and gives objective results that are comparable across the different cell lines and experimental conditions.

Comparison of different cultures demonstrated that cell behavior in the initial period of wound healing is heterogeneous (Figure 2A), and thus, the analysis of wound closure is difficult to be uniform. This is in accord with previous observations (Topman et al., 2012), and thus, the initial period of wound healing was not analyzed further. We found that wound closure with constant speed (linear fit is 0.99) starts for all cell lines tested at the fifth hour after scratching and continues at least up to 10–12 h. After the 12th hour, the number of pioneering cells detached from the monolayer increases, which makes automated analysis less precise (Cortesi et al., 2017), and the velocity of the monolayer decreases for some cell lines (HT1080, A549). Thus, the 6-h window at the 12-h time interval is convenient and sufficient for the detailed analysis.

Our data show that when images were taken with relatively frequent sampling, and the data of several experiments are averaged, the reproducibility of the wound area measurement becomes relatively high—we obtained a small SEM (standard error of mean) and a high *r*^2^ for the linear approximation of the area changes. The results were similar (SEM < CI 95% and *r*^2^ > 0.985) for different cell types—epithelial cells, highly transformed cancer cells, and fibroblasts.

Using the quantitative approach developed, we first analyzed the effect of the substrate on cell motility since different adhesivities might affect wound closure dynamics. Early studies show that decreasing substrate adhesivity accelerates cell motility, but mainly for highly invasive cancer cells (Pavlíková et al., 1998), while increasing adhesivity had an opposite effect (Olbrich et al., 1996). Recent modeling shows that increasing the adhesion results in increased spreading of cells and larger cell speeds (Cao et al., 2019). While all types of cells examined spread faster and better on the plastic surface (data not shown), their motility changed in a different way. Our results show that there is a different effect of a highly adhesive surface on cell motility. Our data show that the influence of substrate adhesiveness on motility is cell-type specific.

Second, we analyzed the effect of anti-microtubule drugs on cell motility. Studies have indicated that drugs able to depolymerize microtubules can suppress the migration of different cell types (Jordan, 2002; Ganguly et al., 2012), except neutrophils where even complete depolymerization of MTs accelerates cell motility (Keller et al., 1984). Our data confirm this conclusion—both types of fibroblasts (3T3 and HPF) became immotile under high dose treatments with MT inhibitors; however, some strongly transformed cells (HeLa, PC-3, U2OS, and HT1080) were found to have residual motility at the level of about 50% of the control.

Inhibition of cell motility by low concentrations of MT inhibitors is more doubtful. The results obtained by different authors (Liao et al., 1995; Grigoriev et al., 1999; Ganguly et al., 2012; Parker et al., 2017) are contradictory, and our data show that inhibitory effect strongly depends on the particular cell line and might depend on the drug used. Low doses of nocodazole and Taxol inhibit the motility of NRK fibroblasts completely at concentrations of 400 and 100 nM, respectively (Liao et al., 1995). Using the Vero cell line, we observed a subtle decrease in cell motility under the treatments with nanomolar doses of nocodazole (170 nM), Taxol (50 nM), and vinblastine (50 nM) and complete abrogation of motility under higher doses (1.7 μM of nocodazole and 1 μM of Taxol) (Grigoriev et al., 1999). From the results obtained in the current study on different cultures, it is not possible to see any common effect for anti-microtubule drugs in the nanomolar range of concentrations.

Non-monotonic curves of the cells’ Transwell migration efficiency change with respect to the drug concentration for nocodazole and Taxol treatments in the range of concentrations 10 pM to 300 nM were described for HeLa and CHO cells (Ganguly et al., 2012)—authors report decreased motility of cells under the action of 1- to 10-nM drug doses and restoration of cell motility under the action of drugs sufficient to depolymerize MTs. Our data based on the wide concentration range (10–1,000 nM) might represent a similar effect—we observed increased motility for HPF, A549, HeLa, and HT1080 cells, yet we never saw inhibition of motility by the lower doses of anti-MT drugs (data not shown). Thus, explanation on stimulation of cell migration in the absence of MTs suggested by Ganguly et al. (2012) might represent only Transwell assay and hardly can be generalized for the 2D motility. We also see that effects of micromolar concentrations of drugs depolymerizing and stabilizing MTs are cell line dependent and, in some cases (MCF-7 cells), drug dependent. These differences require further elucidation.

## Conclusion

Our results on quantitative analysis of the wound healing process allow us to state the following.

The time window in the range of 4–12 h after scratch is sufficient to evaluate drug effect on the wound healing process. During this period, wound closure is well characterized by a single parameter—slope of the curve of area change. To achieve a good linear fit, 10 min frequency sampling and data averaging are needed.

Higher adhesivity of the substrate is preferable for wound healing analysis because it accelerates cell spreading and gives a homogenous monolayer. The role of cell division in the closure process is negligible.

The effects of MT inhibitors on the wound healing process could be summarized as follows:

(i) Cell motility for some cells is accelerated by nanomolar doses of MT inhibitors, which may explain increased metastasis for low-dose treatment by Taxol (Volk-Draper et al., 2014; Li et al., 2016).
(ii) Motility inhibition at minimal mitostatic doses for fibroblasts and cancer cells is always low.
(iii) There is a large difference in the slowdown effect induced by the inhibitors in micromolar doses between cell lines. It is notable that all cancer cells in our study showed a certain level of tolerance to drug treatment, while normal cells are more sensitive.
(iv) Some cell lines demonstrate a specific response on one inhibitor and significantly a smaller response on another inhibitor. Since the effects of all inhibitors on the dynamic instability of MTs are the same, we suggest that a significant slowdown of cell motility by low doses is induced not only by the interaction of inhibitors with MTs but there are more specific mechanisms that require further elucidation.

## Data Availability Statement

The raw data supporting the conclusions of this article will be made available by the authors, without undue reservation.

## Author Contributions

SK performed all benchwork: cell culture maintenance, experimental conduction, microscopy recording, data collection, and data analysis and interpretation, also the development of the MatLab tool. Current work is considered to be a significant part of the author’s Ph.D. thesis. The manuscript was prepared by SK. AU performed MatLab code optimization, assisted in code adaptation for image filtering core, and provided feedback and discussion on the statistical tools to be applied. IV provided a problem statement to fulfill the gap of the knowledge; mentored the experiments and data collection; performed the peer-review and data-quality evaluation, mentoring on data interpretation, and interpretation of results; provided guidance to the composition of Introduction, Results, and Discussion; and performed a pre-submission peer-review of the manuscript. All authors contributed to the article and approved the submitted version.

## Conflict of Interest

The authors declare that the research was conducted in the absence of any commercial or financial relationships that could be construed as a potential conflict of interest.
