# Supplementary material for: The Frequent Sampling of Wound Scratch Assay Reveals the “Opportunity” Window for Quantitative Evaluation of Cell Motility-Impeding Drugs
Source: Front Cell Dev Biol. 2021 Mar 11;9:640972. doi: 10.3389/fcell.2021.640972 (PMC7991799; doi:10.3389/fcell.2021.640972)
Supplement: Supplementary file 1 [file Table_1.DOCX]

**The example of microtubule inhibitors dilution for wound healing assay (for Nocodazole)**

The dry Nocodazole must be completely diluted in DMSO to make a primary 50 mM stock. This solution is a primary stock and need to be aliquoted and must be stored at – 30 C.

The secondary stock solution is diluted in DMSO to final concentration of 1 mM or 1000 µM of Nocodazole and stored at – 30 C too.

**The dilution in a culture media (for cultivation or CO-2 independent media for live cell imaging)**

The highest concentration we used in experiment was 3000 nM. To make 1 mL of working solution drug we added 3 µL of DMSO working solution to 997 µL of CO2 independent DMEM. This solution contains ~0.3% DMSO and considered to show no unrelated toxicity. This solution then diluted by 3x step down to make a descending range of concentrations: 1000 nM, 300 nM, 100 nM, 30 nM, 10 nM, 3 nM. To keep the concentration precision the dilution must be performed according to Chart.

Note that at every step, the final concentration of DMSO in media is decreased 3 times (0.1% of DMSO for 1000 nM dose, 0.03% of DMSO for 300 nM etc.). So, the control is DMSO free or must be repeated in same order to track the differences.

Chart for performing serial dilution of drugs.

| Concentration | Volume of DMEM | Volume to transfer / Volume of |  |
| --- | --- | --- | --- |
| 3000 nM | Initial stock of 1000 uL | 300 uL | The initial working solution is mixed with a certain volume of no drug DMEM in a well and the procedure repeated for the next well. The excessive amount of media then removed to make all well contain equal amount of media |
| 1000 nM | 700 uL | 300 uL / 1000 uL |  |
| 300 nM | 600 uL | 300 uL / 900 ul |  |
| 100 nM | 700 uL | 300 uL / 1000 uL |  |
| 30 nM | 600 uL | 300 uL / 900 ul |  |
| 10 nM | 700 uL | 300 uL / 1000 uL |  |
| 3 nM | 600 uL | 300 uL / 900 ul |  |
| … etc |  |  |  |
