# Supplementary material for: The Frequent Sampling of Wound Scratch Assay Reveals the “Opportunity” Window for Quantitative Evaluation of Cell Motility-Impeding Drugs
Source: Front Cell Dev Biol. 2021 Mar 11;9:640972. doi: 10.3389/fcell.2021.640972 (PMC7991799; doi:10.3389/fcell.2021.640972)
Supplement: Supplementary file 2 [file Table_2.DOCX]

The image filtering procedure

Each image in a sequence passes sequential filtering to produce an intermediate image on which edge detection is performed and a binary mask generated.

1. Image smoothing

After initiation, the processing starts with multiplication of input image to itself (image square).

Then difference of two images smoothed by Gaussians was performed. The 1^st^ image was smoothed using radius of 3 pixels for *Σg_1_(x)* and second the radius of 1 pixel for *Σg_2_(x)*:

$$\sum\Delta f\left( x \right)=\sum g_{1}(x)-\sum g_{2}((x)$$

The procedure was performed in MatLab with 2-D “Gaussian” function and it produces a new image *f(x)*.

1. Hybrid filtering

To denoise the image the custom hybrid filter was created. The filter performs snooting of fields of pixels with values close to mean in a certain span. The wound lattice reach for pixel values remains intact while gap is flattened.

The filter utilizes 4 directional scans in a given kernels of size 2k+1 (k_1_, k_2_, k_3_, k_4_) displayed at Figure 1. The k is non-zero odd integer and default value is 5.

Kernels constructed as smoothing masks with linear pattern:

$$k_{1}=\left[ \begin{matrix} 0 & 0 & \begin{matrix} 0 & \begin{matrix} 0 & 0 \end{matrix} \end{matrix} \\ 0 & 0 & \begin{matrix} 0 & 0 & 0 \end{matrix} \\ \begin{matrix} 1 \\ 0 \\ 0 \end{matrix} & \begin{matrix} 1 \\ 0 \\ 0 \end{matrix} & \begin{matrix} 1 & 1 & 1 \\ 0 & 0 & 0 \\ 0 & 0 & 0 \end{matrix} \end{matrix} \right] , k_{2}=\left[ \begin{matrix} 0 & 0 & \begin{matrix} 1 & \begin{matrix} 0 & 0 \end{matrix} \end{matrix} \\ 0 & 0 & \begin{matrix} 1 & 0 & 0 \end{matrix} \\ \begin{matrix} 0 \\ 0 \\ 0 \end{matrix} & \begin{matrix} 0 \\ 0 \\ 0 \end{matrix} & \begin{matrix} 1 & 0 & 0 \\ 1 & 0 & 0 \\ 1 & 0 & 0 \end{matrix} \end{matrix} \right]$$

$$k_{3}=\left[ \begin{matrix} 1 & 0 & \begin{matrix} 0 & \begin{matrix} 0 & 0 \end{matrix} \end{matrix} \\ 0 & 1 & \begin{matrix} 0 & 0 & 0 \end{matrix} \\ \begin{matrix} 0 \\ 0 \\ 0 \end{matrix} & \begin{matrix} 0 \\ 0 \\ 0 \end{matrix} & \begin{matrix} 1 & 0 & 0 \\ 0 & 1 & 0 \\ 0 & 0 & 1 \end{matrix} \end{matrix} \right] , k_{4}=\left[ \begin{matrix} 0 & 0 & \begin{matrix} 0 & \begin{matrix} 0 & 1 \end{matrix} \end{matrix} \\ 0 & 0 & \begin{matrix} 0 & 1 & 0 \end{matrix} \\ \begin{matrix} 0 \\ 0 \\ 1 \end{matrix} & \begin{matrix} 0 \\ 1 \\ 0 \end{matrix} & \begin{matrix} 1 & 0 & 0 \\ 0 & 0 & 0 \\ 0 & 0 & 0 \end{matrix} \end{matrix} \right] ,$$

Each kernel is applied on input image *f(x)* and produces sub-image *f_kn_(x)*,

$$f_{kn}\left( x \right)=\left\{ \begin{aligned} \begin{matrix} f_{k1}(x)=f\left( x \right)*k1; \\ f_{k2}(x)=f\left( x \right)*k2; \end{matrix} \\ f_{k3}(x)=f\left( x \right)*k3; \\ f_{k4}(x)=f\left( x \right)*k4; \end{aligned} \right.$$

to be processed further. Then pixel with minimal value in each position of the image is determined among *f_kn_(x).* These minimal values are used to generate product image *g(x)*.

$$g\left( x \right)= \sum_{i=1}^{n} \min\left( f_{kn}\left( x \right) \right),$$

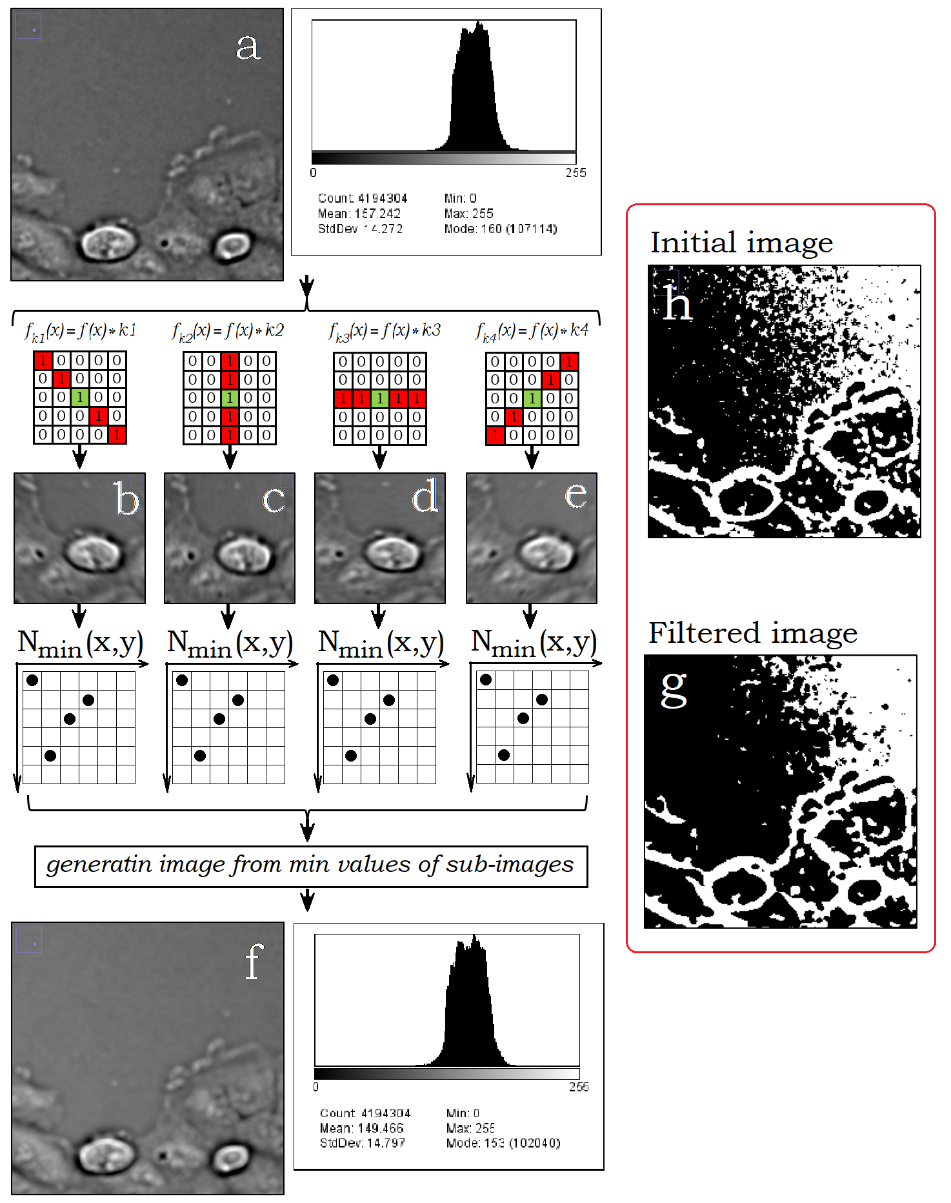


Figure 1. Smoothing hybrid filter implementation: input image (a), rich for noises (h) goes to parallel convolution with 4 kernels to produce (b-e) intermediate sub-images. Then, minimum values used to create output image(f). Its histogram remains largely the same, however the number of small objects significantly decreased(g).

1. Histogram reconstruction

At the next step image histogram reconstruction performed to divide the wound and gap. The “TopHat” filtering procedure is applied to the image g(x) to decrease structures of background. The TopHat filter is 2-D MatLab filter with kernel of 13x13 pixel and magnitude *H=1* was applied:

$\omega\left( k \right)=A\left( H\left( x-a \right)-H\left( x-b \right) \right),$

and $g^{'}\left( x \right)$ convolution product image was obtained (Fig. 3E).

$g^{'}\left( x \right)=\frac{1}{N\times M}\sum_{i=1}^{n} \sum_{i=}^{n} \omega(k)*(g(x))$,

After it, the image averaging (smoothing) procedure was performed using 20x20 pixel kernel averagefilter function to suppress the particles in wound gap.

This function is available at https://www.mathworks.com/matlabcentral/fileexchange/40174-average-filter?s_tid=srchtitle.

The result of this sequential cascade is presented at Figure 2


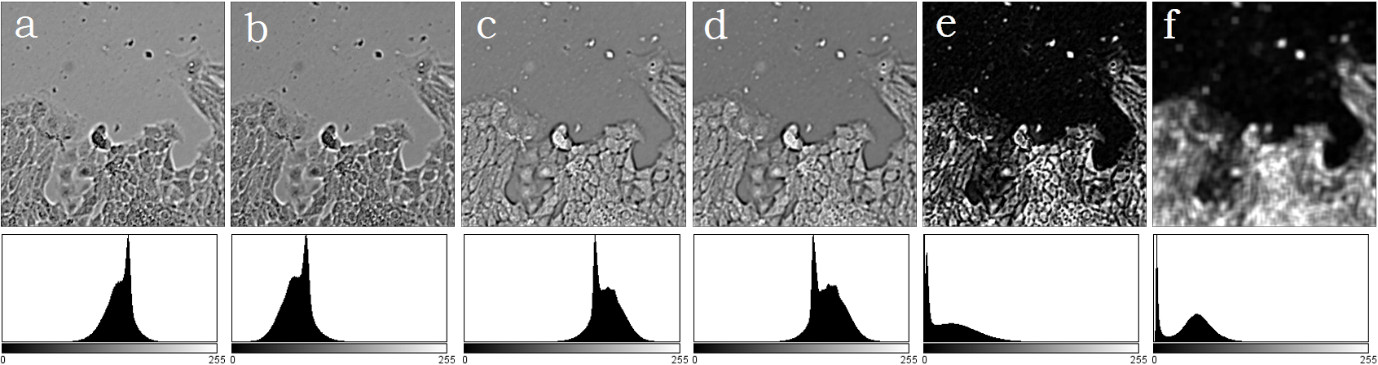


Figure 2. The image histogram transformation: a) original input image; b) result of image powering; c) difference of Gaussians smoothing; d) denoise hybrid filtering; e) background subtraction by ”TopHat” filter; f) smoothing by averaging;

1. Defining thresholds to create binary image.

The segmentation for determining wound area starts with defining the thresholds to binarize image.

Grayscale image *g’(x)* obtained at the previous step (Figure 2, f) was normalized and binarized by *mean-*based threshold algorithm.

Thus, threshold *T* is set as fraction of *arithmetical mean,* obtained as,

$T=\frac{1}{n}\sum g^{'}\left( x \right)/a$,

where *a* is coefficient considered to be positive integer and set equal to *π*. Thus, by binarization we distinguish most pixels of cell monolayer and gap (Fig 4 a).

The homogenous empty gap pixels generally have values in the left part of histogram and since they occupy not more than 1/3 of the area these pixels are below the *arithmetical mean*. The majority of pixels representing cell monolayer have values above the mean (Figure 3), forming the second peak of histogram. There is certain fraction of pixels sharing values with gap pixels at the edge of the wound.


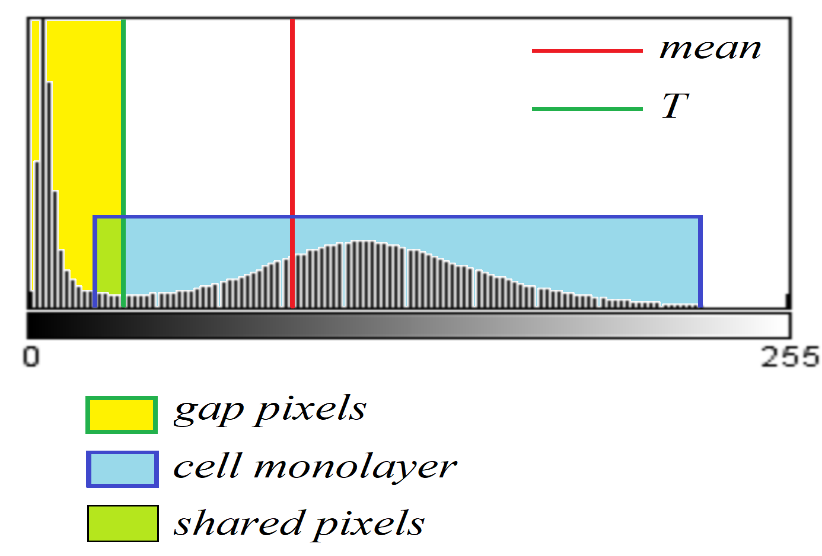


Figure 3. The reconstructed histogram of image

1. Conditional operator loop to control the binarization quality.

After binarization the gap pixels value become 1 and cell monolayer become 0. However, there may be 2 possible output when binary image appear inverted (the gap become 0 and cell monolayer 1). It is an artifact caused by reconfiguration of images with poor quality. To correct this artifact, we apply the Conditional operator to check and fix the proper segmentation of the gap by defining the pixel value in a geometrical center of the image. It is expected there is always a small cleft (unclosed area of the wound) in a center of image.

The procedure contains a logical loop to check whether the count of pixels in the kernel 11*11 located in the image center is below 11, and if is not, the image then inverted. Then it performs “fill holes” and check the condition if image contains both 0 and 1 pixels. If not, the image doesn’t meet this condition and loop restores the previous step and apply threshold obtained as image mean,

$$T=\frac{1}{n}\sum g^{'}\left( x \right)$$

This weight provide f the gap pixels gives sufficient elevation of the mean value for correct segmentation of bad images. Schematic representation of conditional operator and result given at Figure 4.


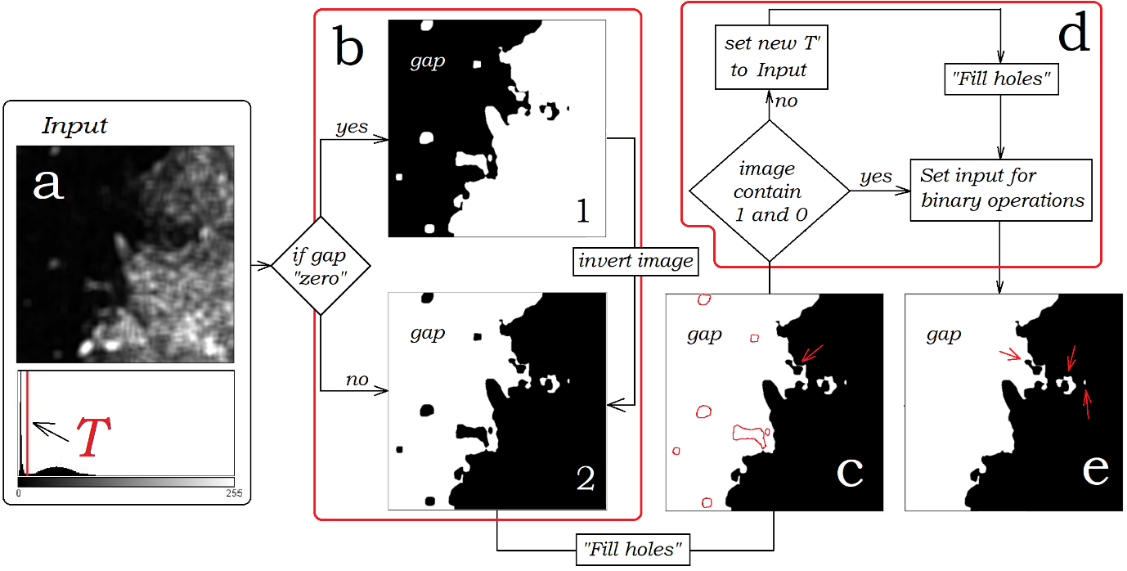


Figure 4. The conditional operator loops: a) input with histogram and threshold (red line), b) possible variant of image binarization and in conditional operator loop, c) imaged passed first *Conditional operator* loop; d) second loop of conditional operator; e) image assigned as input for operations with binary images.

1. Smoothing operations with binary image

Then binary smoothing procedure applied to obtained binary image to remove to obtain solid perimeter line of wound gap.

Further operations are performed with several structuring elements represented at Figure 5, with an intermediate result. The goal is to wipe away bottlenecks and isthmuses at a wound edge (Figure 5 a, red arrows on enlargement). These segmentation steps of MatLab script consist of sequential 1) *binary closure, 2) dilatation, 3) closure* and 4) *erosion*.


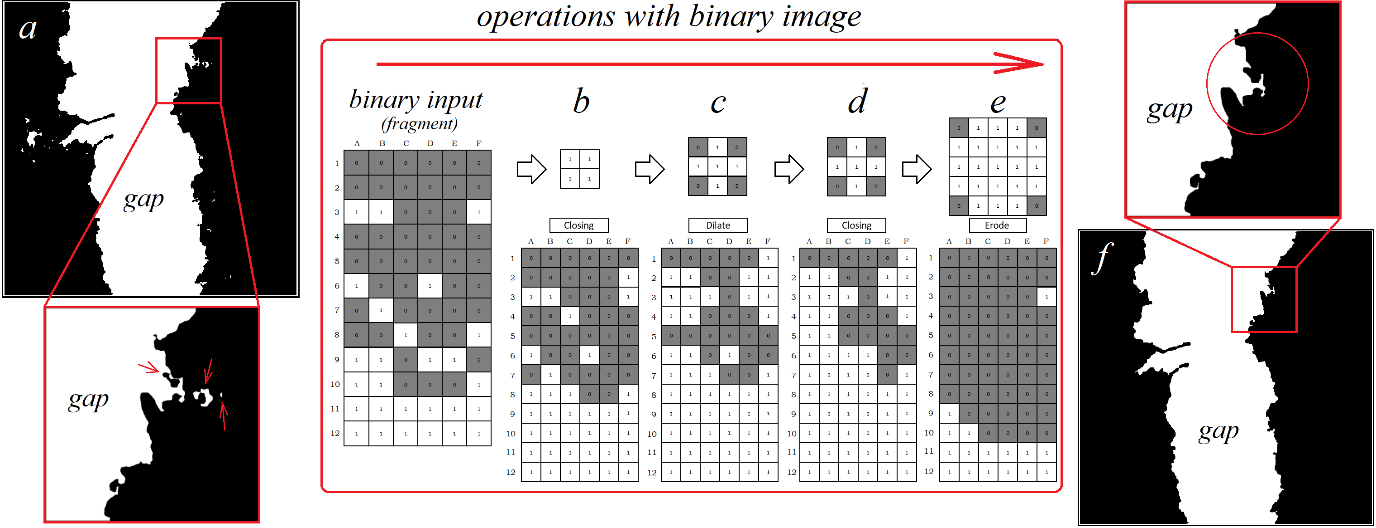


Figure 5. Obtaining binary mask of wound gap: 1) on a binary input(b) we apply closure (A) with 2x2 structuring element to connect pixels on the boundaries of the gap; 2) binary dilatation (B) for 1-pixel structuring element to connect occasionally lost pixels from previous step(pixel at 6A); 3) Binary closure with 1-pixel structuring element (C) applied to connect omitted pixels on the boundaries of the wound; 4) Binary erode with 3-pixel structuring element (D) to offset the previously occasionally added pixels.

The sequence of smoothing operations removes unconnected pixels at the wound edge and connect pixels related to the wound gap as it is shown at Figure 5a (arrows on enlargement) and 5f (arrowed and circled enlargement). This binary mask is used for the measurements the pixel counts representing gap area.
