# Supplementary material for: The Frequent Sampling of Wound Scratch Assay Reveals the “Opportunity” Window for Quantitative Evaluation of Cell Motility-Impeding Drugs
Source: Front Cell Dev Biol. 2021 Mar 11;9:640972. doi: 10.3389/fcell.2021.640972 (PMC7991799; doi:10.3389/fcell.2021.640972)
Supplement: Supplementary file 3 [file Table_3.DOCX]

**List of software used to perform the study and prepare the manuscript**

1. The authors used MatLab &Simulink based on Campus-Wide license. Script was tested to be compatible with MatLab2014b and older.

Current version is stable for MatLab2020b with next toolboxes:

MATLAB Version 9.9 (R2020b)

Simulink Version 10.2 (R2020b)

Computer Vision Toolbox Version 9.3 (R2020b)

GPU Coder Version 2.0 (R2020b)

Image Processing Toolbox Version 11.2 (R2020b)

MATLAB Coder Version 5.1 (R2020b)

MATLAB Compiler Version 8.1 (R2020b)

Mapping Toolbox Version 5.0 (R2020b)

Parallel Computing Toolbox Version 7.3 (R2020b)

Signal Processing Toolbox Version 8.5 (R2020b)

Simulink Check Version 5.0 (R2020b)

Simulink Code Inspector Version 3.7 (R2020b)

Simulink Coder Version 9.4 (R2020b)

Simulink Compiler Version 1.1 (R2020b)

Statistics and Machine Learning Toolbox Version 12.0 (R2020b)

Symbolic Math Toolbox Version 8.6 (R2020b)

MatLab can be downloaded from <https://www.mathworks.com/products/matlab.html>

2. Fiji is an image processing package, bundling a lot of plugins of ImageJ which facilitate scientific image analysis.

The stable version of FIJI/ImageJ can be downloaded here <https://downloads.imagej.net/fiji/latest/fiji-win64.zip>

3. Krita is a free and open-source application. You are free to study, modify, and distribute Krita under GNU GPL v3 license <https://krita.org/en/download/krita-desktop/>

The GraphPad is software to perform statistical analysis and graphing solution for scientific research and can be downloaded here https://www.graphpad.com/scientific-software/prism/
